# Strange attractors: purpose, synchronicity, flow, and the neurodynamics of meaning

**DOI:** 10.1093/nc/niag063

**Published:** 2026-10-19

**Authors:** Steven Kotler, Michael Mannino, Karl Friston, Scott Kelso

**Affiliations:** Flow Research Collective, United States; Center for Complex Systems and Brain Sciences, Florida Atlantic University, 777 Glades Road, Boca Raton, FL 33431, United States; Flow Research Collective, United States; Center for Complex Systems and Brain Sciences, Florida Atlantic University, 777 Glades Road, Boca Raton, FL 33431, United States; Miami Dade College, 300 NE 2nd Ave, Miami, FL 33132 Miami, United States; University College London, London, Gower Street, London, WC1E 6BT, UK; Center for Complex Systems and Brain Sciences, Florida Atlantic University, 777 Glades Road, Boca Raton, FL 33431, United States; Bath Institute for the Augmented Human, University of Bath, Claverton Down, Bath BA2 7AY, United Kingdom; Intelligent Systems Research Centre, Ulster University, Derry/Londonderry, Magee Campus, Londonderry BT48 7JL, Northern Ireland, United Kingdom

**Keywords:** consciousness, metastability, active inference, coordination dynamics, flow, predictive processing

## Abstract

This paper proposes a neurodynamic framework for meaningful experience, grounded in the recursive alignment of brain dynamics across time. We argue that the meaningful experiences known as “purpose”, “synchronicity”, and “flow” are not distinct phenomena, but temporal expressions of the same underlying process: metastable coherence in the brain’s attractor landscape. Synchronicity marks transient realignments across representational levels; flow reflects the low-entropy navigation of emergent structures; and purpose encodes long-term integration into causal agency of a self-model. Drawing on active inference, dynamical systems theory, and recent work on predictive processing and memory consolidation, we formalize these states as trajectories through phase-space—differentiated by temporal depth, stability, and precision-weighting. We offer empirical predictions for each regime, linking neural signatures such as sharp-wave ripples, P300b activity, and network coherence to experiential markers of meaning. We also propose a developmental interpretation informed by Robert Kegan’s subject–object theory, framing purpose as an evolving attractor shaped through recursive narrative and identity updating. This model suggests that meaning is not only symbolic or semantic, but dynamic, embodied, and experimentally testable: a measurable function of the brain’s capacity to coordinate across nested timescales.

*We dedicate this paper to the memory of Steven Bressler, a wonderful scientist, husband, father, mentor, and friend, who made great strides in the field of neuroscience and complex systems. Among Steven’s academic achievements were mentoring numerous successful PhD students, making significant original contributions to computational neuroscience, directing the Center for Complex Systems and Brain Sciences, and being recognized by his peers for naming the “gamma wave” with his own mentor Walter Freeman!*

## Introduction

What does it mean to experience synchronicity, the sensation of meaningful coincidence (Jung 1960), as if life events have aligned with one’s private mythos? What does it mean to be swept up by purpose and feel drawn to something larger, older, and more enduring than the self—a sense of ontological security often associated with narrative identity (McAdams and McLean 2013; Singer et al. 2013)? And what occurs, neurobiologically, when these sensations of coherence become embodied as effortless effort, deep absorption, and skilled action, the hallmarks of the state of consciousness known as flow (Csikszentmihalyi 1990; Dietrich 2004)?

Synchronicity, as we use the term here, refers to the felt experience of meaningful coincidence—the sudden convergence of internal states and external events in a way that seems improbably significant, yet lacks causal explanation. You think of someone you have not spoken to in years, and the phone rings. A book falls off a shelf, seemingly at random, and answers a question with which you have been silently struggling. These are the moments people often colloquially describe as “too weird to be just coincidence”. In popular usage, synchronicity is often taken as a sign or message, an uncanny alignment between the inner world of thought and feeling and the outer world of events. It is typically interpreted through a symbolic and, often, a mystical lens. What gives these moments their psychological charge is not the (apparent) objective rarity of the event, but the subjective sense of meaning that arises from it. Synchronicity, then, is less about what happens in the world and more about how the mind makes sense of it—a realignment of disparate elements into a coherent, emotionally resonant whole.

Likewise, purpose, as we use the term here, refers to the felt sense of long-range coherence, i.e. a psychological alignment between one’s identity, agency, values, and deeply held goals that persists across time. Rather than a set of abstract principles, purpose, experientially, is a lived architecture of meaning, where predictions about who we are and where we are going become integrated into our sense of self and guide behavior with minimal ongoing deliberation. Purpose emerges when temporally deep goal structures—those that extend over months, years, or even decades—become synchronized with the neural systems that instantiate the self. In this state, actions feel not just worthwhile in the moment but woven into a broader narrative arc that anchors motivation and orients decision-making. However, we note that purpose may act on a different timescale than agency. For example, Sloan et al. (2023) found that agency in infants can emerge abruptly, as a punctuated phase transition in a coupled brain–body–environment system, often unfolding over seconds to minutes as a functional relation is discovered and stabilized. These agentive realizations were marked by sudden changes in coordination patterns, exploratory pauses, and anticipatory action, indicating short-timescale reorganization rather than enduring goal commitment. Purpose, by contrast, reflects the slow consolidation of such agentive episodes into temporally extended narratives of selfhood, where repeated experiences of effective action are integrated across contexts and time. In this view, agency provides the moment-to-moment discovery of “I can”, whereas purpose reflects the long-range stabilization of “this is who I am and where I am going”, operating over a deeper temporal horizon than the dynamics that give rise to agency itself.

Finally, flow refers to a state of consciousness in which attention becomes fully absorbed in a challenging yet manageable activity, producing a seamless merging of action and awareness (Kotler et al. 2022). In flow, conscious representations of sensory inputs and outputs are tightly coupled to largely nonconscious, automatic processes honed through experience (Kotler et al. 2025). Self-conscious evaluation recedes, time perception often distorts, and performance feels both effortless and highly effective, because, from a theoretical perspective (see below) prediction errors vanish, and are continuously integrated into a high-bandwidth perception–action loop. Flow emerges in the narrow channel where skill and challenge are precisely matched, sustaining engagement while stretching abilities enough to foster growth. Neurocognitively, flow reflects a metastable balance between stability and flexibility, maintained by dynamic coordination across brain networks supporting attention, reward, and sensorimotor integration (Hancock et al. 2025). Precision-weighted updates keep attention locked to immediate task demands while allowing rapid adaptation to changing conditions. Within this regime, habitual mental actions, honed through extensive experience, rapidly select and update belief states in response to contextual cues, while temporally “shallow” yet highly precise planning keeps attention locked to immediate task demands. Prediction errors are not eliminated but absorbed into the ongoing action–perception loop, guiding adaptive adjustments without interrupting the experience. Here, the self is not deliberating about its goals but embodying them, integrating information across sensory domains to maintain control in volatile environments (Kotler et al. 2025). What defines flow is less the external nature of the task than the internal organization of experience, where all cognitive and emotional resources align toward the unfolding moment, yielding deep absorption, intrinsic motivation, and a heightened sense of agency.

Although these three phenomena are typically treated as distinct experiences—one philosophical, one psychological, and one neurocognitive—we argue that they can be understood as temporal expressions of a shared process: large-scale metastable coordination across distributed brain networks (Tognoli et  2007; Deco and Kringelbach 2017; Tognoli and Kelso 2014). This account builds upon our previous work exploring the role of metastability in consciousness, intuition, and flow (Kotler et al. 2022, 2025; Solms 2021), and extends the framework into a new explanatory domain: the neurodynamics of meaning, by proposing a unified model rooted in metastable coordination and a precision-weighted attractor landscape (Kelso 2025).^1^

Traditionally, each phenomenon has been studied in isolation. Purpose is often framed in narrative psychology as the integration of temporally extended goals and values into a coherent autobiographical self-model (Singer 2004; McAdams and McLean 2013). Synchronicity, in contrast, has remained outside the scientific mainstream, associated with depth psychology, and often dismissed as a form of apophenia or patternicity (Brugger 2001; Shermer 2008). Flow, while more empirically grounded, is typically explained in terms of task-related variables such as challenge-skill balance, task-specific attention, and immediate feedback (Nakamura and Csikszentmihalyi 2002).

We propose a unifying neurodynamic account. Purpose emerges when the metastable self-model becomes phase-locked with temporally deep, goal-related priors encoded in higher-order predictive hierarchies (Pezzulo et al. 2015; Friston 2010). Synchronicity arises when multiple high-salience attractors—such as unresolved personal problems, emotionally charged symbols, and long term goals—are co-activated and temporarily stabilized in dynamic coordination (Buzsáki 2015a, 2015b; Tognoli and Kelso 2014). Flow is the embodied correlate: a precision-weighted, prediction-fulfilled crossing of integrated attractor states that is distinguished by reduced surprise and enhanced control (Friston et al. 2012a, 2012b, 2012c; Ulrich et al. 2016; Kotler et al. 2022).

We argue these are not independent experiences but rather constitute the same large-scale mechanism operating at different temporal scales. This recursive architecture likely involves the medial prefrontal cortex and midline default-mode structures, which instantiate the self across temporal scales and are crucial for integrating interoceptive, autobiographical, and goal-related information (Northoff 2013, 2016). Purpose reflects metastability over months or years; synchronicity, over seconds to minutes; and flow, over minutes to hours. Each involves the same core processes: attractor alignment, uncertainty reduction, and large-scale integration that links the self-model to sensorimotor loops, goal-directed networks, and salience detection (Deco et al. 2011; Northoff 2013; Friston et al. 2012a, 2012b, 2012c).

In the sections that follow, we synthesize evidence from systems neuroscience, active inference (Friston 2010), dopaminergic precision-weighting (Friston et al. 2009), and sharp-wave ripples (SWRs) implicated in path navigation and memory consolidation (Buzsáki 2015a, 2015b; Jadhav et al. 2012). We show how attractor landscapes can realign into experiences of deep meaning that reflect the brain’s capacity to detect, stabilize, and act upon configurations of high epistemic and affective salience—patterns that convey significance, shape behavior, and update the self-model.

## Theoretical foundations

### Anchoring meaning: from dynamical significance to symbolic form

Before exploring the neurodynamics of meaning, we should say plainly what we mean by the term. We use “meaning” to mean felt-significance: the salience, self-relevance, and emotional weight that make certain experiences matter to the organism having them (Freeman 2000). In a neurodynamical sense, meaning lives in the shape of the attractor landscape—in how steeply that landscape pulls the system toward some paths and not others, making certain trajectories feel compelling and worth pursuing. This is not a representation the brain computes and hands off; it is a property of how the system’s dynamics are organized. Meaning, on this view, is dynamical and embodied before it is symbolic or computational.

That emphasis might seem to put us at odds with the substantial body of work that treats symbolic and semantic meaning rigorously in terms of brain dynamics (Tabor 2009; beim Graben and Potthast 2012; beim Graben et al. 2019). It does not. Felt and symbolic meaning are the same landscape, viewed at different resolutions. The brain never encounters raw, undivided possibility; it always groups similar states into working categories—clusters of slightly different brain states the system treats as equivalent when they persist over time (Atmanspacher and beim Graben 2009). A symbol is an especially stable and reusable version of one of these groupings. It persists long enough that the brain can label it, return to it, and manipulate it as a discrete unit (Tabor 2009; beim Graben and Potthast 2012). What separates something felt from something symbolic, then, is the stability of the underlying pattern. As we will see, this same property—how deep and durable a pattern becomes—helps distinguish a fleeting synchronicity from an enduring sense of purpose, and determines whether a pattern remains a passing experience or consolidates into a symbol the mind can reuse (Mondal 2022, 2024).

### Metastability and information dynamics

Any account that treats synchronicity, flow, and purpose as temporally-distinct expressions of the same neuronal process must first explain how large-scale neural systems coordinate flexibly across time. Metastability provides this foundational mechanism. Metastability refers to the dynamic balance between integration and segregation in neural systems (Kelso 1995). In a metastable brain, individual regions maintain partial autonomy while also participating in transient, coordinated assemblies. This enables rapid shifts between functional states, balancing global coherence with local specialization.

The dynamic nature of a metastable system is well captured by coordination dynamics. Imagine two neural populations that influence one another but operate at different rhythms. At times, their activity becomes coordinated; at others, it drifts apart. Their relationship—described by their “relative phase”—never settles permanently into one pattern. If the mismatch between their rhythms increases, stable coordination can disappear altogether through a saddle-node bifurcation. Yet, traces of those stable states remain. The system’s trajectory slows and lingers near these “remnants”, as if drawn toward a coordination pattern that no longer fully exists, before moving on (Kelso 1995, 2012; Tognoli and Kelso 2014; Hancock et al. 2025). The result is neither full synchrony nor complete independence, but a restless back-and-forth: periods of near-coordination (“dwelling”) interrupted by moments of separation (“escaping”), a pattern known in coordination dynamics as “relative coordination”. Two tendencies coexist without either winning out: a pull toward integration and a pull toward independence. Metastability is this unresolved tug-of-war (Bressler and Kelso 2001; Kelso 2012).

This tug-of-war explains the term’s second half: “stability”. Because a metastable system does not settle down and instead keeps visiting and leaving different configurations, it has a wide range of possible states and the flexibility to move freely among them—a balance thought to be a hallmark of healthy brain function (Deco et al. 2011; Hancock et al. 2025). Too much integration collapses this range of states and, when taken to the extreme, shows up as the runaway synchrony of a seizure. On the other side, too much segregation blocks the large-scale binding that unified experience depends on. It is important, however, to distinguish metastability from criticality, a related idea with which it is often confused. Metastability describes how much a system’s coordination pattern varies over time; criticality describes brief, scale-free bursts of activity that peak right at a transition point (Hancock et al. 2025). The two can co-exist—when the weight given to prior beliefs is balanced against the weight given to incoming sensory evidence, free energy is minimized and neural activity shows a slowing similar to self-organized criticality—yet they are not the same thing. Our account rests on metastable coordination, not on criticality by itself.

In earlier work on the hard problem of consciousness, we built on Kelso and colleagues’ identification of metastability as the defining feature of coordination dynamics in complex systems, including the brain (Bressler and Kelso 2001, 2016; Kelso 1995, 2001a, 2001b, 2012; Tognoli and Kelso 2014). We proposed that subjective experience emerges when neural activity enters a regime of sufficient coherence, integration, and temporal persistence, allowing information to become phenomenally accessible—that is, experienced as qualia—rather than merely processed.

Prior work has also linked metastable dynamics to spontaneous thought, creative cognition, and the emergence of intuition and insight (Kotler et al. 2025; Deco and Kringelbach 2017). Here, we argue that the deep sense of meaning behind purpose, synchronicity, and flow arises from similar metastable dynamics: a coordination between the brain systems that handle self, salience, needs, and goals. We also note that recent empirical findings are consistent with this claim (Hancock et al. 2026; Eldaly et al. 2026). The remainder of this section develops this proposal, situating metastable coordination within active inference, grounding it in the reactivation mechanisms that reshape the attractor landscape offline, and connecting it to Freeman’s account of how meaning arises from population-level neural dynamics.

### Active inference, priors, and precision

We begin by placing metastable coordination dynamics within Karl Friston’s active inference framework, clarifying how long-range priors, precision weighting, and hierarchical self-models give rise to the experience of purpose as a temporally extended, goal-directed mode of sense-making. In Friston’s framework, agents attempt to minimize variational free energy, seeking to reduce uncertainty about the causes of their sensory input by updating internal models or sampling new data (Friston 2010; Friston et al. 2012a, 2012b, 2012c; Parr and Friston 2017, 2018; Peters et al. 2017). These models are organized hierarchically, with high-level priors encoding long-range expectations about the self and the world (Clark 2013; Pezzulo et al. 2015; Hohwy 2016).

In this model, purpose can be understood as the alignment of high-level goal priors with the active self-model. When the system is confined to a metastable regime where top-down predictions about identity, value, and long-term goals align with bottom-up sensory input and feedback, the agent experiences coherence—a directional, teleological sense of being (Friston 2018).

Neuromodulators such as dopamine and norepinephrine regulate the precision weighting of these predictions. Dopamine encodes the expected precision of action policies—a structured plan of action across time—effectively gating which intentions are expressed (Friston et al. 2012a, 2012b, 2012c; Schwartenbeck et al. 2015). Norepinephrine signals unexpected uncertainty and adjusts the system’s volatility (Yu and Dayan 2005). In this view, these systems arbitrate competing courses of action, both shaping behavior and producing the sense of agency characteristic of synchronicity and flow.

Together, these systems enable flexible inference across scales. They allow metastable states where high-level priors, salient environmental inputs, and embodied action harmonize into a unified attractor experienced subjectively as clarity, direction, and meaningful engagement. In the context of flow, this gating mechanism also facilitates the seamless execution of hierarchically aligned movement policies. Here, precision-weighted predictions guide real-time action with minimal prediction error across sensorimotor loops, resulting in a fluency of action and the sense of control that are characteristic of the state (Friston et al. 2012a, 2012b, 2012c; Pezzulo et al. 2015; Schwartenbeck et al. 2015; Kotler et al. 2022).

### Sharp-wave ripples and opportunistic assimilation

While metastability and precision-weighted inference help explain how meaning arises during experiences of synchronicity, flow, or purpose, the formation of these attractor alignments—particularly during moments of rest or incubation—requires memory reactivation and pathfinding mechanisms. This brings us to SWRs, the hippocampal micro-events that drive the spontaneous reconfiguration of the attractor landscape (Buzsáki and Moser 2013; Kotler et al. 2025). SWRs are transient, high-frequency oscillatory events that originate in the hippocampus, and are associated with memory consolidation, imagination, and planning. Research suggests SWRs support goal-directed behavior, replaying potential paths through an attractor landscape in order to predict future trajectories. This process may privilege affectively charged or self-relevant attractors, allowing the brain to “test fit” potential resolutions to narrative discontinuities or unresolved goals, while extending the architecture of meaning beyond immediate experience.

When multiple attractors coactivate during SWR replay and become transiently phase-locked, prior work showed that the result can be intuitive insight (Kotler et al. 2025). Here, we extend that mechanism to include synchronicity, proposing that synchronicity emerges when SWRs trigger the co-activation of previously unlinked representations—i.e. a personal problem, a symbolic image, and an environmental cue. If these representations become phase-locked in a metastable burst, the system registers the event as a signal of global coherence. Importantly, these ripple-driven activations are not confined to the hippocampus. They synchronize with prefrontal cortices, especially ventromedial prefrontal cortex (vmPFC), whose activity has been shown to amplify the evaluative salience of contextually relevant stimuli, effectively turning neutral inputs into meaningful cues (Jadhav et al. 2012; Buzsáki 2015a, 2015b). This process lends flow, synchronicity and purpose their felt sense of significance, while providing a mechanism for what Seifert et al. (1995) described as “opportunistic assimilation”, a process of sudden insight wherein latent problems are reactivated and resolved by the appearance of a contextual trigger.

Importantly, we distinguish meaningful pattern recognition—such as opportunistic assimilation, synchronicity, purpose, and flow—from false positives, including apophenia and pareidolia, that are often described as patternicity (Shermer 2008). The distinction lies in the minimization of prediction error. In false pattern recognition, *aberrant precision weighting* leads the brain to assign excessive confidence either to top-down priors or noisy sensory inputs. When priors are overweighted, internally generated expectations dominate perception, producing hallucination, delusion, or conspiracy ideation (Corlett et al. 2007). When noisy sensory input is over-weighted, the system may misinterpret randomness as salience (Friston 2009; Fletcher and Frith 2009). Unlike false pattern detection, *synchronicity* reduces uncertainty across multiple hierarchical levels of the model. These events produce an adaptive update that minimizes free energy while increasing coherence between internal models and external inputs. They are characterized not by epistemic distortion, but by integrative reorganization and informational efficiency (Friston et al. 2017a, 2017b; Clark 2013).

### Freeman’s intentional neurodynamics and the dynamical basis of meaning

To bring these ideas together, we turn to Walter Freeman’s theory of intentional neurodynamics, the framework within which the other theories in this paper are situated. Freeman’s work located meaning at the mesoscopic scale: in the collective activity of neural populations, not in the spike trains of single cells (Freeman 1997, 2000, 2007; Mannino and Bressler 2018; Kozma and Noack 2017). Freeman saw meaning as something the brain constructs, through recursive cycles of action and perception, rather than something it decodes from the world. His central construct is “intentionality”. Freeman used the term biologically, tracing it to Thomas Aquinas: the goal-directed thrusting of the body outward into its environment. Intentionality names the property our account requires: a system whose dynamics reflect the significance of things for the organism. This significance emerges from the organization of neuronal populations and not from symbolic representation. In our account, this becomes the tendency of a metastable system to arrange its attractor landscape around what is salient and self-relevant.

Freeman’s theory is organized as a hierarchy of what he termed “K-sets”, which span scales from local to global and micro to macro (Skarda and Freeman 1987; Kozma and Freeman 2009; Mannino and Bressler 2018). At the lowest level, a K0 set is a population of non-interacting neurons that are either all excitatory or all inhibitory. KI couples such sets together. KII mixes excitatory and inhibitory populations under negative feedback, which generates the gamma-range limit-cycle oscillations whose spatially coherent envelope is called the “wave packet”. KIII sets assemble multiple KII units into a full sensory system, producing the chaotic dynamics that support learning and memory. KIV integrates several KIII sets across a brain hemisphere, giving rise to intentionality.

The K-sets hierarchy tells us how neuronal populations are organized across scales. To describe how these populations behave collectively, dynamical systems theory uses an “order parameter”: a macroscopic variable that captures the coordinated state of the system. For Freeman, this order parameter is the wave packet’s spatial pattern of amplitude modulation, or SAM: the contour of amplitude across the generating population. He understood this pattern as the manifestation of an attractor laid down through learning (Freeman 2000). Coordination dynamics, by contrast, takes relative phase as its order parameter (Kelso and Tognoli 2009; Bressler and Kelso 2016; Bressler et al. 2018; Mannino and Bressler 2018).

Both approaches rest on the nonlinear coupling of neural oscillators, invoke broken symmetry, and appeal to Haken’s concept of “circular causality”: the idea that the coordinated activity among many neurons gives rise to a collective variable that, in turn, constrains the very neurons that created it. A “phase transition” is the moment when the neurons spontaneously organizes into a new spatial pattern. Such transitions can be carried and described by either by the relative phase or by the amplitude of oscillation, making Freeman’s and Kelso’s accounts complementary rather than competing (Kelso and Tognoli 2009).

These two approaches are not identical, however. Freeman emphasized the succession of learned attractors that emerge as the brain interacts with the world. Kelso emphasized the metastable coordination that allows the brain to move flexibly among such patterns without becoming locked into any one of them. Freeman focused on amplitude as the signature of meaning, where Kelso focused on relative phase as the signature of coordination. Together, they describe complementary aspects of the same metastable dynamics (Kelso and Tognoli 2009; Mannino and Bressler 2018).

These shared metastable dynamics are expressed in Freeman’s theory as continual movement through an evolving attractor landscape. Cortical activity continually creates, dissolves, and revisits attractor states that are globally stable yet locally unstable (Skarda and Freeman 1987; Freeman 2000). Rather than remaining fixed within a single attractor, activity briefly stabilizes into a wave-packet frame, dwells, then escapes into the next. This same pattern has also been described as chaotic itinerancy**:** an orbit that wanders among the remnants of attractors rather than settling into any one of them (Tsuda 2001, 2013; Bressler and Kelso 2016). Freeman’s cinematographic hypothesis gives this continual reorganization temporal structure, proposing that theta oscillations organize a succession of wave-packet frames, each expressing a transient attractor before yielding to the next (Freeman 2007).

Where do the hippocampal events of the preceding discussion enter this architecture? Freeman located the substrate of intentionality in the limbic system, and gave context to what he called the “space–time loop”, in which hippocampus and entorhinal cortex exchange integrated activity with the sensory cortices to supply the spatial and temporal context from which meaning is built (Mannino and Bressler 2018). SWRs are, in the account developed here, the offline operation of exactly this loop: transient hippocampal reactivations that replay and recombine candidate trajectories, reshaping the curvature of the KIV landscape between one action-perception cycle and the next (Buzsáki 2015a, 2015b; Jadhav et al. 2012; Kotler et al. 2025). Ripple-driven reactivation, then, is what Freeman modeled as the limbic contribution to the landscape’s form: it reweights which attractors are available to the next frame. Buzsáki’s mechanism supplies the neurophysiology of Freeman’s space–time loop.

The remaining framework, active inference, was anticipated by Freeman, yet not named. His account of intentionality is predictive: the organism generates a hypothesis about how the world will respond to its action, acts to test it, and learns from the sensory consequence. Reafference, the reshaping of perception by self-generated movement, binds the action to its sensory return (Freeman 2000). In variational terms, this cycle is active inference: action selected to fulfill predictions and minimize surprise across a hierarchical model (Friston et al. 2017a, 2017b; Friston and Frith 2015; Parr and Friston 2019; Friston et al. 2024).

Where the two coordination programs meet, symmetry-breaking marks the transition from multiple competing possibilities into a single coordinated state. (Kelso and Tognoli 2009). The free energy principle describes this same transition probabilistically: a prior distribution is updated into a posterior through precision-weighted inference.

Freeman’s chaotic attractor landscape is globally stable but locally unstable. A free-energy formulation reads this same landscape as a statistical manifold whose curvature encodes precision. On this reading, the edge-of-chaos traversal of KIV may be understood as motion down free-energy gradients. Freeman’s signature event, when input destabilizes the current attractor and triggers a phase transition into a new AM pattern, can be viewed as a precision-weighted belief update.

Intentionality and free-energy minimization are thus one fact seen from two vantages. Mesoscopically, a body thrusts outward to make the world confirm its model. Variationally, that same system descends a gradient of surprise.

Taken together, these correspondences tell us that within Freeman’s K-set architecture, Kelso’s metastability names the coordination regime in which the ladder operates, Buzsáki’s SWRs supply the space–time loop that reshapes the landscape offline, and Friston’s free energy names the quantity the whole system descends (see Fig. 2). These three descriptions are of a single intentional-neurodynamic process, differing in the order parameter each foregrounds and the timescale over which it is read. Purpose, synchronicity, and flow follow as regimes of this one system: purpose as the slow sculpting of the KIV landscape by consolidated, temporally deep priors that scaffold the self-model; synchronicity as a ripple-triggered phase transition binding previously unlinked attractors into a transiently coherent wave-packet frame; and flow as the sustained, precision-matched traversal of a deep basin through the full action-perception cycle. Each represents a different depth and tempo of the same descent into meaning: a claim that becomes testable once we ask how such large-scale reorganizations announce themselves as measurable brain events, which is the task of the next section.

### P300b, dopamine, and large-scale integration

If meaning emerges through the rapid reconfiguration of metastable attractor landscapes—as Freeman suggests—then moments of successful reorganization should be detectable not only in mesoscopic population dynamics, but also as more global belief-updating events. The system must register the new configuration at a level accessible to conscious awareness and action selection. In active inference terms, this corresponds to a sudden increase in model evidence: uncertainty is reduced across multiple hierarchical levels, and a new prediction regime becomes temporarily stabilized.

The P300b is a likely candidate. It is an event-related potential linked to evidence accumulation, belief updating, and meaningful insight (Polich 2007). In this context, the P300b may serve as the neuronal correlate of Seifert’s opportunistic assimilation (Seifert et al. 1995), signaling the large-scale integration of co-activated attractors into a new belief state. Converging evidence suggests that such integrative events engage vmPFC circuits involved in evaluative weighting, attaching subjective significance to previously neutral cues (Tang and Posner 2009; Jadhav et al. 2012). When these co-activated attractors successfully reduce uncertainty across hierarchical levels, dopamine is released as a precision-weighted update that stabilizes the novel attractor configuration in memory and action (Friston et al. 2012a, 2012b, 2012c).

Crucially, integration is not limited to externally driven task engagement, but can occur when flow begins, when synchronicity registers as meaningful coincidence, and over longer horizons, when purpose is consolidated through repeated episodes of successful alignment. In all three cases, the nervous system must mark the transition*—*“something has changed”*—*and systems should update accordingly.

In the case of synchronicity, the reorganization reflects the co-activation of multiple internally-generated attractors—often affectively charged and involving autobiographical memory, unresolved goals, and symbolic representations—into a coherent interpretive frame. In flow, salience detection triggers a global reorganization similar to synchronicity, but the integration is expressed through action rather than interpretation. Instead of reorganizing symbolic or autobiographical meaning, the system aligns perception, motor output, and task affordances into a unified control state. This action-centered reconfiguration still constitutes a large-scale belief update, and is therefore marked by dopaminergic precision-weighting and P300b activity (Kotler et al. 2022; Polich 2007; Friston et al. 2012a, 2012b, 2012c).

A clarification is helpful here. In treating the P300b and phasic dopamine as neural signatures of these transitions, we do not mean that a meaningful experience is an evoked potential or a burst of neuromodulation. Purpose, synchronicity, and flow are large-scale, emergent modes of coordination. The markers we invoke are readouts of that coordination: indices that a system-wide belief update has occurred and been stabilized. The P300b indexes this integration.

We flag this explicitly because a risk grows as the phenomena become more temporally extended: mistaking a convenient scalp or circuit-level correlate for the phenomenon itself. This risk is least serious for synchronicity, which is genuinely event-like. It is most serious for purpose, which unfolds over months and years and can only ever be partially indexed by any single evoked response. Throughout, then, these markers should be read as empirical windows into the underlying metastable dynamics, not the dynamics themselves.

Over time, those integrative episodes that generalize across contexts and remain predictive are retained, progressively reshaping high-level priors. In this way, purpose reflects the gradual consolidation of lessons learned through repeated moments of coherence (McAdams and McLean 2013; Pezzulo et al. 2015; Friston 2018).^2^

## Tripartite phenomenology as a single dynamical process

Purpose, synchronicity, and flow are traditionally treated as distinct experiences, studied in separate disciplines, explained by different models, and unfolding across different timescales. Yet from a neurodynamic perspective, they appear to be the same core process expressed at varying temporal and representational depths. In this section, we examine each phenomenon in turn—beginning with the slowest and most enduring: purpose.

### Purpose

Purpose is often conceived as a high-level cognitive construct: a narrative identity or coherent autobiographical arc about who we are and where we are going (McAdams and McLean 2013; Singer et al. 2013). While psychologically compelling, this view does not explain how such narratives become felt experience: stable, motivating, and embodied. We propose that purpose is best understood as a metastable attractor landscape in which the self-model becomes phase-locked with hierarchically deep goal priors (Northoff 2016; Friston 2018).

These high-level priors are temporally rich—they encode not just immediate expectations but long-range predictive structures that span months, years, or decades. Beyond propositional goals or semantic beliefs, these priors function as motivational attractors that minimize long-term uncertainty about one’s place in the world, values, and contribution. When these attractors become stably integrated with the self, agents experience a sense of purpose: directionality, coherence, and existential salience (Hill and Turiano 2014). This framing aligns with empirical findings that purpose functions as a systemic buffer—correlating with lower inflammatory markers, reduced allostatic load, and increased physiological resilience over time (Zilioli et al. 2015).

Neurobiologically, this integration may be marked by increased coherence between default mode structures (e.g. mPFC, PCC), frontoparietal control systems, and midbrain dopaminergic circuits (Seeley et al. 2007; Shine et al. 2019a, 2019b; Schwartenbeck et al. 2015; Friston et al. 2012a, 2012b, 2012c). The sustained tonic activity of the dopaminergic system seems to act as a precision-weighting stabilizer**,** increasing confidence in the long-range attractor structure (Schwartenbeck et al. 2015; Friston et al. 2012a, 2012b, 2012c). This coherence would allow temporally deep priors to guide behavior without constant reevaluation—freeing working memory and attentional bandwidth for adaptive engagement while preserving motivation.

Subjectively, this state feels deeply meaningful. Not simply that an action is worth doing, but that it is woven into the structure of self across time. Purpose is a dynamical binding: a metastable fusion of identity, intention, and expectation that persists across perturbations and self-updates. Once established, purpose reshapes perception and action by filtering salience, guiding exploratory behavior, and amplifying learning signals around long-range goals. This model resonates with Barrett’s theory of constructed emotion, which frames affective meaning as emerging from the brain’s interoceptive predictions and categorization processes (Barrett 2017).

### Synchronicity

In contrast to purpose, synchronicity is a transient phenomenon that occurs when disparate attractors are co-activated and held in dynamic suspension. This suspension enables rapid, high-precision inference, making these co-activated inputs feel meaningfully related, even in the absence of causal linkage (Jung 1976; Main 2007).

From a dynamical perspective, synchronicity is the sudden coherence of a metastable pattern involving self, intention, memory, and external inputs. The system recognizes a new higher-order regularity—an emergent coherence across previously unlinked dimensions. This experience is often marked by heightened salience, emotional arousal, and a felt sense of significance disproportionate to the immediate stimulus, but—viewed through predictive-coding—informationally rich (Varela et al. 1991; Kelso 1995; Friston 2017).

Neurologically, we hypothesize a cascade involving sharp wave ripples (Jadhav et al. 2012; Buzsáki 2015a, 2015b), P300b activation, and phasic dopamine release (Polich 2007; Friston et al. 2012a, 2012b, 2012c) that work to simplify models and compress entropy—explaining more with less—by transiently aligning priors across multiple domains. This framing echoes Tschantz et al. (2020), who describe “epistemic foraging” as a form of action-oriented model compression, in which agents update internal models by seeking maximal information gain. Similarly, Clark (2013) emphasizes that cognitive salience emerges when precision-weighted surprise reorganizes hierarchical predictions, allowing the brain to detect novel coherence with minimal computational cost.

In phenomenological terms, what feels like “fate” may be the brain’s recognition of a temporally deep alignment—a hidden structure suddenly made explicit through multiscale inference. In this sense, synchronicity is a form of spontaneous narrative reorganization, a transient, metastable pattern in which identity, value, and external context briefly phase-lock into uncertainty-reducing salience (Seth and Friston 2016; Gallagher 2020). The felt sense of this experience is less a confirmation of belief and more the stabilization of a new attractor configuration that biases future prediction, action, and meaning-making.

### Flow

Flow is the action-phase of this same process. Rather than reflecting the interpretation of meaning, as in synchronicity, or the long-term stabilization of meaning, as in purpose, in flow, this metastable alignment is enacted through embodied control. Perception, action, and intention become tightly coupled, allowing the system to maintain coherence while continuously sampling and responding to changing conditions. Understood through the lens of the free energy principle, prediction errors are minimized through high-bandwidth sensorimotor coupling, and attention is finely tuned to resolve uncertainty in empirically measurable ways (Friston et al. 2017a, 2017b).

Flow has a well-characterized and remarkably consistent phenomenology. Attention becomes hyper-focused and task-specific; action and awareness merge, such that the doing and the sensing of the doing are no longer experienced as separate; self-reflective cognition and awareness of bodily processes recede; time perception is distorted; performance feels at once effortless and acutely controlled; and the activity becomes autotelic, worth doing for its own sake (Csikszentmihalyi 1990; Kotler et al. 2022). These are facets of a single systemic reorganization: when a skilled agent is precisely matched to a tractable challenge, the system settles into a low-surprise regime in which control is maintained without recourse to explicit self-representation (Kotler et al. 2025). The apparent paradox of effortless effort—of performance that is simultaneously high-control and unforced—dissolves once it is read this way, as the deliberative monitoring one’s own action is replaced by the fluency of well-fitted prediction.

In *Surfing Uncertainty*, Clark (2015) argues that cognition is a rolling prediction about the world, modulated by the precision-weighting of incoming errors. In flow, the precision-weighting system appears to reach an “optimal grip”, a term borrowed from Merleau-Ponty and later reformulated into active inference terms by Bruineberg et al. (2018). With optimal grip, there is a highly-efficient attunement between body and world, wherein agency and action become seamlessly integrated (Gibson and Rader 1979; Kelso 2016). Similarly, Tschantz et al. (2020) model exploratory behavior as the emergent outcome of agents minimizing expected free energy. Flow appears to be the phenomenological correlate of these processes: a temporary stabilization of the agent’s trajectory in entropy-reducing action space.

The recession of self-referential processing deserves particular emphasis, because it is where flow’s phenomenology reveals the most about its underlying dynamics (Hancock et al. 2026). In an earlier account of flow’s neurobiology, we argued that this diminished sense of self reflects a localized down-regulation of the medial PFC (mPFC)—a core hub of the default mode network heavily implicated in self-referential processing—rather than a global shutdown of the prefrontal cortex or the default mode network (Kotler et al. 2022). Consistent with this, flow’s phenomenological components appear dissociable: time distortion and the vanishing of self can occur separately and likely reflect different mechanisms. Altered salience-network activity and phasic dopamine appear to shape time perception, while the attenuation of the narrative self follows from localized mPFC hypofrontality (Kotler et al. 2022). What remains in flow, then, is a transformed sense of self: the agent becomes so absorbed in the cycle of prediction and action that the boundary between doer and task blurs (Kotler et al. 2025; Parvizi-Wayne et al. 2024).

Recent work on nondual awareness offers an interesting comparison. Josipovic argues that contemplative practice reduces the habitual partition between subject and object through altered interactions between default-mode and task-positive networks (Josipovic 2014). While flow and meditation differ in important respects, both involve a temporary attenuation of narrative self-processing, consistent with the metastable reorganization proposed here.

Taken together, these three phenomena—purpose, synchronicity, and flow—reflect a single process: metastable alignment across multiple timescales. Purpose unfolds over months to years, flow over minutes to hours, and synchronicity over seconds to minutes.

## An integrated neurodynamic architecture and empirical predictions

The preceding sections elaborated on a single claim: that purpose, synchronicity, and flow do not have three underlying distinct mechanisms but instead are one intentional-neurodynamic system observed in three (temporal) regimes. What remains is unification: to show how one architecture realizes all three experiences and what predictions follow from their shared machinery. In coordination dynamics a “control parameter” is the quantity that, when varied, leads a system through its transitions (Kelso 1995, 2012; Bressler and Kelso 2016). Here, there are four relevant control parameters: the “temporal depth” of the priors engaged, from the seconds of a synchronistic experience to the decades of a purposeful life; the “depth and stability” of the attractor basin the system occupies, from a shallow well quickly escaped to a manifold that persists through perturbation; the “precision balance” struck between prior and sensory contributions to free energy; and the cortical “spatial reach” of the coordinating assembly, from a transient cross-cortical coalition to the hemisphere-spanning integration that Freeman located at KIV.

Read this way, the three regimes line up along the same axis as the machinery the previous section unified: the K-set hierarchy, the ripple-driven space–time loop, and the descent of the free-energy landscape. Synchronicity sits at the shallowest temporal depth: a fast, ripple-triggered phase transition, akin to a Hopf bifurcation, in which normally unlinked attractors are briefly captured into a coherent wave-packet frame under high transient precision before the system itinerates onward (Jadhav et al. 2012; Buzsáki 2015a, 2015b; Kelso 1995; Deco and Kringelbach 2017). Flow sits at an intermediate depth: not the entry into a shallow basin but the sustained traversal of a deep one, with precision matched between prediction and sensory return so that the action-perception cycle runs at what we earlier called optimal grip, held there by locus-coeruleus–noradrenergic gain modulation and the rapid reconfiguration of salience, goal, and executive networks (Aston-Jones and Cohen 2005; Shine et al. 2019a, 2019b; Kotler et al. 2022; Bruineberg et al. 2018; Sandved-Smith et al. 2021). Purpose sits at the deepest level: the slow drift of the KIV landscape itself, as consolidated priors resculpt basin structure over months and years (McAdams and McLean 2013; Pezzulo et al. 2015). These three regimes can therefore be understood as different settings of the same metastable coordination process, distinguished primarily by temporal depth, attractor stability, spatial reach, and precision weighting (see Fig. 1).

**Figure 1:**
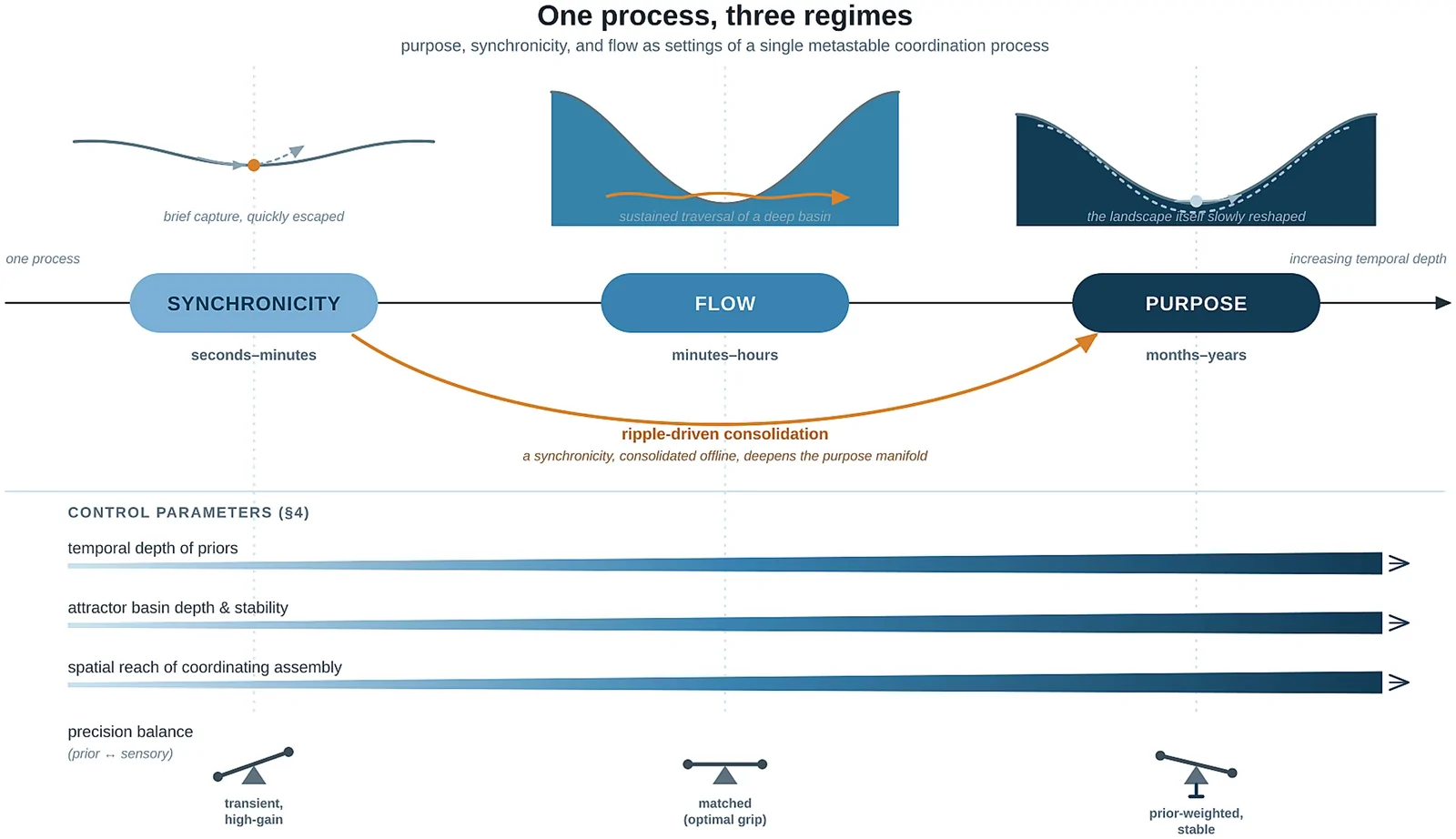
One process, three regimes. Purpose, synchronicity, and flow appear here as three settings of a single metastable coordination process, read from left to right across widening temporal depth—the seconds-to-minutes of a synchronistic convergence, the minutes-to-hours of a flow episode, and the months-to-years over which a purpose consolidates. The upper band traces the same dynamics as a shifting attractor landscape: a shallow, briefly occupied well from which the system quickly escapes (synchronicity), a single deep basin traversed and sustained (flow), and a broad manifold whose contours are themselves slowly resculpted (purpose). Below, three of the control parameters that govern the architecture deepen monotonically along the continuum—the temporal depth of the priors in play, the depth and stability of the occupied basin, and the spatial reach of the coordinating assembly—while precision balance shifts in kind rather than degree, from the transient, high-gain weighting of a momentary convergence, through the matched grip of optimal flow, to the prior-weighted stability that lets a purpose hold its shape. The warm arc marks the one cross-connection drawn directly on the figure: the ripple-driven consolidation by which a synchronicity, replayed and stabilized offline, deepens the very manifold a purpose later inhabits—the fast and slow regimes shown as two ends of one process. Two further couplings run beneath the scheme without being drawn: the shared axis along which default-mode and task-positive systems trade dominance across all three regimes, and the precision misfire that places pathological salience as an immediate neighbor of veridical convergence.

**Figure 2:**
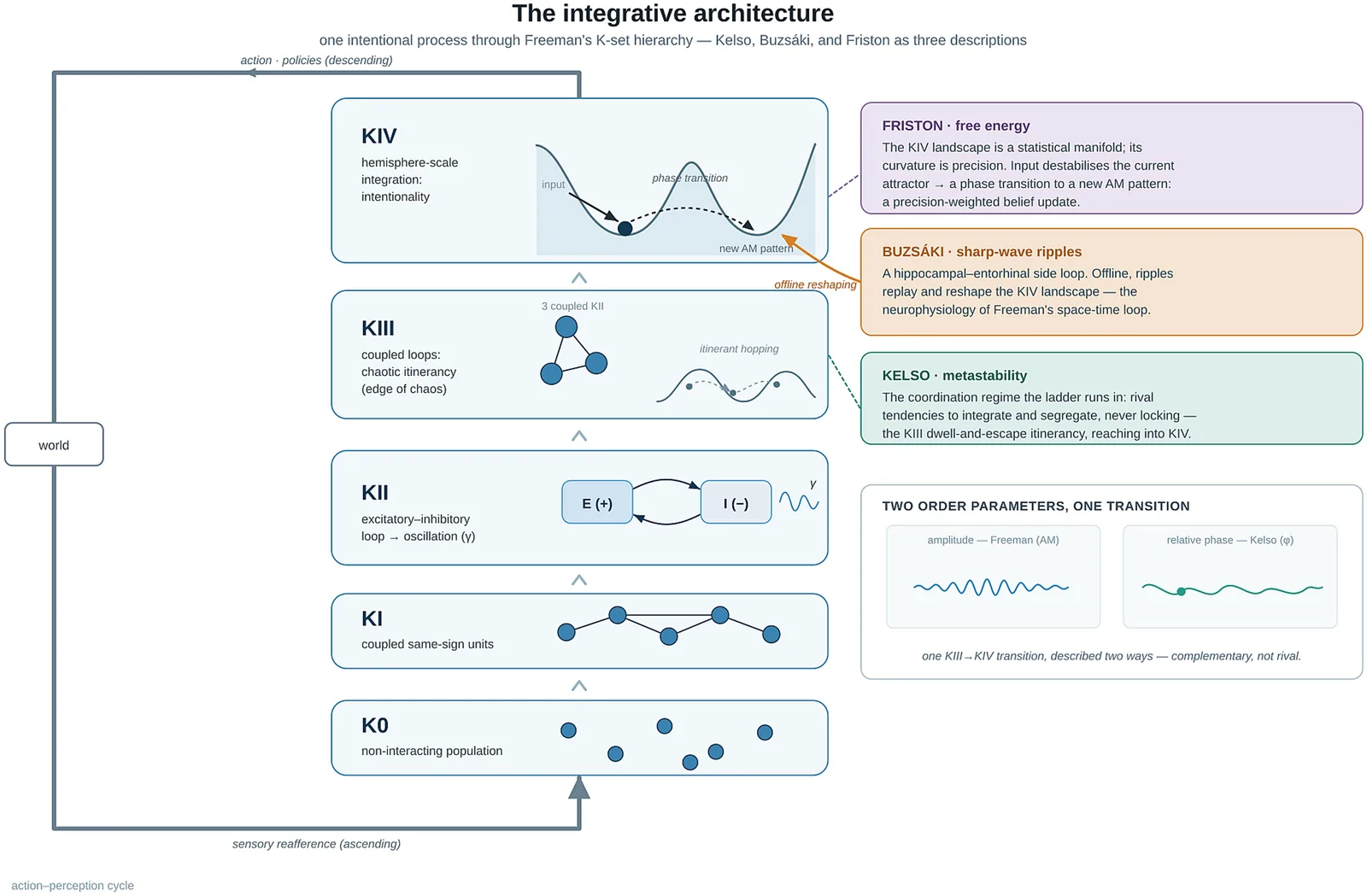
The integrative architecture. A single intentional process is read upward through Freeman’s K-set hierarchy, each level adding scale and integration: non-interacting units (K0) become coupled populations (KI), reciprocal excitatory and inhibitory populations close the feedback loop that sustains oscillation (KII), ensembles of such loops wander among basins in chaotic itinerancy at the edge of chaos (KIII), and hemisphere-scale coordination issues in the global amplitude-modulation patterns the paper reads as intentionality proper (KIV). Onto this one scaffold the three neurodynamic idioms are mapped as three descriptions of a single event. Kelso’s metastability names the coordination regime the ladder runs in—rival tendencies to integrate and segregate that never lock—most visible as the dwell-and-escape itinerancy of KIII reaching up into KIV. Friston’s free energy names the quantity the ladder descends, the KIV landscape now a statistical manifold whose curvature is precision, so that Freeman’s signature transition—Input destabilizes the reigning attractor and the field jumps to a new AM pattern—Becomes a precision-weighted belief update. Buzsáki’s SWRs supply the neurophysiology of Freeman’s space–time loop, a hippocampal–entorhinal side circuit that, offline, replays and reshapes the KIV landscape a purpose will later occupy. The whole hierarchy sits within the action–perception cycle of active inference, action descending toward the world and sensory reafference ascending to be explained away. The inset makes the complementarity explicit: Freeman’s amplitude and Kelso’s relative phase are two readouts of one KIII-to-KIV transition, coordinate descriptions of a single passage.

The cross-connections between these regimes are constitutive, not incidental. The sharp-wave ripple that seeds a synchronicity is the same event that, repeated and consolidated offline, deepens the purpose manifold—so the fast and slow regimes are two ends of one consolidation process. And the coordination between default-mode and task-positive systems forms a single shared axis whose sign and timescale distinguish the regimes: reduced anticorrelation as the narrative self-attenuates in flow, transient co-activation as unlinked contents bind in synchronicity, and slow directional coupling as the self-model is scaffolded into purpose (Northoff 2013, 2016; Josipovic 2014).

Because the regimes are settings of shared parameters, their empirical signatures are best stated as single markers read across all three, which is also where the account becomes falsifiable. Consider belief-updating markers first. The P300b and ripple-triggered evoked responses should appear in all three regimes, but scaled to the depth of the update and timed to its timescale. In synchronicity, which is genuinely a discrete transition, these responses should be most event-like and scalp-detectable. In flow, they should be elevated but folded into the ongoing action loop. In purpose, as the previous section cautioned, no single evoked response can capture the update; it should be only partially and cumulatively indexable (Polich 2007; Friston et al. 2012a, 2012b, 2012c).

In line with this proposed architecture, large-scale network signatures should behave similarly across all three temporal regimes. We predict hippocampal–mPFC–DMN coupling for synchronicity, reduced default-mode/task-positive anticorrelation with salience-executive reconfiguration for flow, and slow mPFC and posterior-cingulate drift for purpose. As these are expressions of a single prediction, the same midline hubs should participate throughout, while the sign and timescale of their coupling discriminates the three regimes (Seeley et al. 2007; Shine et al. 2019a, 2019b; Northoff 2016).

The same pattern should appear in Information-theoretic measures. We expect transient spikes in mutual information during synchronicity, efficient, low-entropy processing with high, task-relevant signal-to-noise during flow; and a long-range decline in prediction-error cost as priors consolidate into purpose. Neurophysiologically, flow should also be accompanied by elevated beta coherence in sensorimotor loops (Ulrich et al. 2016; Hancock et al. 2026). Behaviorally and experientially, we expect a felt significance disproportionate to the eliciting stimulus in synchronicity, smoother kinematics and reduced reaction-time variability in flow, and reportable directionality and coherence of the narrative self in purpose.

Three predictions follow directly from this framework. First, greater attractor plasticity should facilitate all three phenomena together rather than selectively enhancing one. Second, experimentally shifting any of the control parameters should predictably move phenomenology along the continuum. For instance, increasing the temporal depth of engaged priors should transform the transient coherence characteristic of synchronicity into the more stable dynamics of flow or purpose. Third, because these markers index the same underlying quantity: the reduction of variational free energy and the accrual of model evidence, they should co-vary across phenomena (Friston et al. 2017a, 2017b; Friston and Frith 2015).

## Conclusion

The framework we have outlined reframes meaning not as a property of objects or events, but as a dynamic configuration of brain states—a recursive loop of pattern recognition, action and integrative processes. Within this loop, synchronicity provides the epistemic spark, flow the embodied execution, and purpose the long-term convergence. Together, they form a self-organizing grammar for coherence. This account suggests that what we call “meaningful experience” is a form of real-time model optimization, enacted under conditions of high novelty and sufficient internal readiness. Synchronicity is not supernatural, but supra-predictive: it reflects moments when previously independent attractors become temporarily aligned, producing high salience and surprise. Flow, meanwhile, is the low-surprisal traversal of these new structures, while purpose is the integration of those patterns into the generative model over time. Crucially, this architecture offers a neurobiological basis for why meaning feels urgent, directional, and emotionally charged. It is not a projection. It is a readout of system-wide coherence—a temporary window into the integration of identity, goal, action, and world.

This view also clarifies the developmental role of insight, novelty, and intentional practice. If meaning is metastability made conscious, then we can support this architecture’s formation by designing environments that maximize informational richness without overwhelming cognitive load, and training attention to detect, stabilize, and enact emerging attractor patterns.

Additionally, while the primary focus of this model is explanatory and theoretical, we highlight that its clinical relevance is significant. The brain’s capacity for recursive meaning-making, central to experiences of purpose, synchronicity, and flow, can, under certain conditions, become maladaptive. When attractor landscapes are overly plastic or easily perturbed, the system may fall into pathological coherence: excessive attribution of meaning to noise, or destabilization of identity-level attractors. Clinically, this is seen in conditions such as schizophrenia, mania, or dissociation, leading to apophenia, delusion, or narrative fragmentation. Conversely, insufficient metastability may manifest as rigidity or rumination, characteristic of depression or OCD. From a therapeutic standpoint, this model suggests that interventions promoting narrative coherence and top-down attractor stability—such as logotherapy, trauma-informed narrative therapies, or purpose-centered coaching—could be neurodynamically beneficial. Flow-inducing or synchronicity-evoking practices, when appropriately deployed, may reorganize maladaptive attractor patterns by opening windows for belief updating (Carhart-Harris and Friston 2019), emotional integration, and reconstitution of a healthier self-model.

This neurodynamic account echoes Robert Kegan’s theory of adult development, in which growth is marked by transitions from subject to object—where what once governed us becomes visible, held, and integrated into higher-order coherence (Kegan 1982, 1994). In our terms, each developmental shift is a slow bifurcation of the self-model into a deeper attractor manifold, sculpted by recursive episodes of flow, synchronicity, and narrative integration. Purpose, then, becomes both a long-term attractor and an evolving capacity to update identity across nested timescales.

In an age of information overload and meaning collapse, this work offers a hopeful premise: that coherence can be cultivated, purpose can be earned, and even uncanny synchronicities may be the nervous system’s attempt to reduce uncertainty.

## Footnotes

^1^
Technically, this precision-weighted attractor landscape is an attracting manifold, which plays the role of a statistical manifold with an information geometry. Specifically, this statistical manifold can be regarded as a landscape of variational free energy. This free energy landscape provides a nice way of connecting neuronal coordination dynamics with inference and belief updating in the brain: one can interpret such neuronal dynamics as flowing down free energy gradients on the attracting manifold. At the same time, one can interpret the dynamics as a minimization of free energy or a maximization of Bayesian model evidence (a.k.a. marginal likelihood). Because every point on this statistical manifold—of internal states—corresponds to a Bayesian belief about the external causes of the sensorium, neuronal dynamics can be read in a literal sense as Bayesian belief updating. Here, a Bayesian belief corresponds to the conditional probability distribution over external states encoded by the neuronal—i.e. internal—state. Crucially, these Bayesian beliefs are equipped with uncertainty or its complement precision (i.e. confidence). The special aspect of free energy landscapes is that, by construction, their curvature or roughness corresponds to precision. This means that Bayesian beliefs held with undue precision capture neuronal trajectories in steep valleys that may, with learning, canalize themselves (Lin et al. 2017). This speaks to the importance of getting the precision—i.e. shape of the attractor landscape—right. We will see later that the relative precision of sensory or likelihood contributions to free energy has to be balanced against prior beliefs, in order to maintain an authentic grip on the world that is informed by prior experience. From a dynamical systems perspective, this is called balanced synchronization (a.k.a. synchronization of chaos) (St Clair et al. 1990; Hunt et al. 1997; Shew et al. 2011; Friston and Frith 2015; Jafri et al. 2016; Palmer et al. 2019). A numerical simulation of balanced synchronization can be found in (Friston et al. 2012a, 2012b, 2012c). In this example, prior precision was changed, relative to sensory precision, to show that when the two were balanced, free energy was minimized, perceptual categorization was optimized and, interestingly, neuronal dynamics showed characteristic slowing, i.e. a kind of self-organized criticality (Vespignani and Zapperi (1998).

^2^
From the perspective of the free energy principle, one could regard the “purpose attractor” as a “pullback attractor” (i.e. a random dynamical attractor) (Crauel and Flandoli 1994; Wang 2012; Beck and Ramstead 2025). Interestingly, the internal subspace of this pullback attractor corresponds to the attracting manifold that underwrites the Bayesian mechanics of the free energy principle. More generally, the pullback attractor *per se* is often read in terms of “preferred” or “characteristic” states of the generative or world model (Friston et al. 2023), which fits well with the notion of a “purpose” attractor; in other words, any system will look as if it is purposely working towards its preferred or characteristic states.
